# Anorectal Malformations: The Pivotal Role of the Good Clinical Practice

**DOI:** 10.1155/2023/3669723

**Published:** 2023-10-31

**Authors:** Filomena Valentina Paradiso, Sara Silvaroli, Riccardo Rizzo, Lorenzo Nanni

**Affiliations:** Division of Pediatric Surgery, “Agostino Gemelli” University Polyclinic Foundation IRCCS, Rome 00168, Italy

## Abstract

Anorectal malformations (ARM) without a fistula are a rare congenital condition. Although may seem more simple to repair compared with ARM with fistulas, surgery has proved to be challenging. We report the case of a newborn who presented a well-formed anus and normal genitalia; a blind-ending anal canal was detected after the insertion of a rectal probe, thus allowing the diagnosis of ARM. Anal probing straight after birth avoids the possible complications related to intestinal obstruction due to a missed diagnosis of ARM. Examination of the perineal region is an important step in the evaluation of the newborn and represents the tool for a prompt identification of ARM. Adding anal probing to accurate inspection perineum is a good clinical practice and should always be performed even in presence of a normal-looking perineum.

## 1. Introduction

Anorectal malformation (ARM), commonly referred to imperforate anus, is a congenital condition that may go undetected after birth. In most of the cases, a normal anus is missing, and the rectum empties either anteriorly into the perineum or into the vagina in females or into the urinary tract in males. The rarest forms of ARM are represented by the anal stenosis (where an intact funnel-shaped anal canal is present) and ARM without a fistula, where no communication exists between the rectum and the genital or the urinary tract [1]. The perineum should be assessed carefully during the first examination after birth since, in cases of very low ARM, the anal dimple may have a normal appearance, and absence of patency may only be recognized by probing the anus. Due to their rarity, very low forms of ARM without a fistula can massively impact patients' and caregivers' quality of life if not recognized and treated promptly.

## 2. Case Presentation

**Figure 1:**
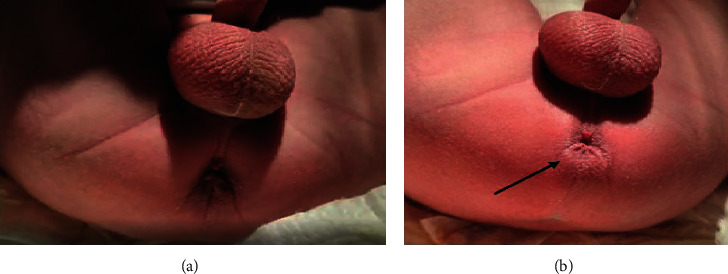
Perineal appearance on the 1^st^ day of life showing a normal-looking anus in a boy (a) and the appearance of a lump (black arrow) in the posterior aspect of the anus revealing a funnel-shaped canal (b).

**Figure 2:**
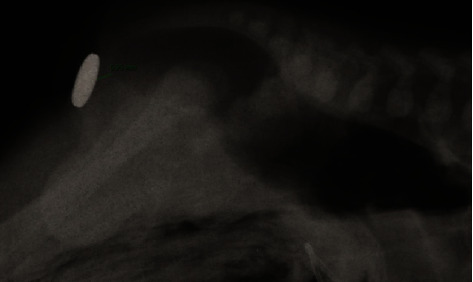
Cross-table lateral radiograph taken at 24 hours of life. A radiopaque marker was placed at the level of the anal dimple.

At birth, a male neonate born at 40 weeks of gestational age with a birth weight of 3820 g (average for gestational age, Z-score 0.7, 75°pc) showed normal buttocks, normal genitalia, and good-looking perineum with a funnel-shaped anus (Figure 1). Prenatal findings were entirely normal. Since the routine attempt to pass a small anal probe was unsuccessful, a nasogastric tube and a central venous catheter were inserted, and clinical observation was started. The patient was kept nihil by mouth for 24 hours, waiting for the passage of meconium. The panel of the studies to exclude associated malformations showed normal results. At 16 hours of life, the patient showed a significant abdominal distension; no passage of meconium or meconuria was observed. During a Valsalva maneuver, a lump was observed in the posterior aspect of the anal dimple. Cross-table lateral X-ray was performed demonstrating a distance of 5 mm between the rectal cul de sac and the skin (Figure 2).

**Figure 3:**
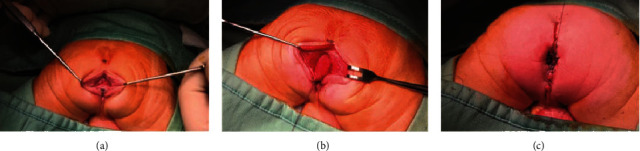
Intraoperative pictures showing the intraoperative findings and the main passages of the posterior-sagittal anorectoplasty (PSARP) performed on the 1^st^ day of life. Incision and rectal cul de sac were found (a), rectal mobilization (b), and neoanus centered in the sphincter complex (c).

On the 2^nd^ day of life, a mini-primary posterior sagittal anorectoplasty (PSARP) was performed (Figure 3), and the intraoperative finding showed the rectal pouch outside from the sphincter muscle as for ARM without a fistula. Postoperative course was uneventful, and the baby was passing meconium on day one and was started on oral feeds by the third day after surgery. The patient was discharged on the 5^th^ postoperative day, and anal calibrations were started after three weeks.

## 3. Discussion

ARM without a fistula is a congenital anomaly in which an imperforate anus is associated with a rectal pouch without a fistula. The sphincter complex usually surrounds the anal dimple.

It occurs in approximately 5% of all cases of ARM, and up to 50% of the cases are associated with trisomy 21 [1, 2]. A multifactorial etiology has been recognized for this condition. ARMs develop at an early gestational age, as a result of an incomplete division of the cloaca from the urorectal septum [3]. Genetic factors can play a central role, as demonstrated in patients affected by trisomy 21 that show an ARM incidence of 2–8% or higher when compared to the general population [4].

In a retrospective study conducted on 1,846 patients with ARM, 1,174 presented other associated defects, being chromosomic anomalies in 11% of the cases [5].

Moreover, maternal exposure to drugs can affect normal gut development. A recent systematic review and meta-analysis demonstrated an association between ARMs and maternal use of antiasthma medications, psychotropic medications, and painkillers [6].

Nowadays, the prenatal diagnosis of ARM is still lacking, and the diagnosis is generally made after birth [7].

In 10% of the cases, patients affected by ARM present at least three of the anomalies defining the VACTERL association: vertebral, anorectal, cardiac, tracheoesophageal, renal, and limbs [8].

Prenatal recognition of VACTERL anomalies may promote earlier diagnosis of ARM, but these data are still discussed [9].

ARM is commonly diagnosed at birth, when an imperforate anus is detected. In very low forms of ARM, the anal dimple may resemble a normal anus, and diagnosis of ARM may go unrecognized. This finding can be particularly confusing in ARM without a fistula where no passage of meconium is observed. When ARM is associated with a well-developed perineum, a delay in diagnosis may ensue leading to signs and symptoms of bowel obstruction. In this condition, an early diagnosis made by anal probing avoids early and long-term complications related to intestinal obstruction (perforation, sepsis, respiratory distress due to abdominal distension, and electrolyte disorders) [10, 11]. Generally, a delayed diagnosis of ARM is defined as a diagnosis made after the first 48 h of life, and it has been reported that the median age at diagnosis of perforation in ARM cases was 48 h [12, 13].

As Turowski et al. reported, one in five neonates born with the imperforate anus had a delayed diagnosis despite the standardized national and international guidelines recommend a routine physical examination of all newborns within the first 48 h of life [11–15].

Thus, especially in the normal looking perineum, a careful inspection followed by anal probing should be mandatory. As in our case, the inability to insert a firm rectal tube into the rectum was the most important tool for diagnosis.

A prompt clinical recognition of ARM based on the Krickenbeck classification [16] allows the clinicians to start a comprehensive preoperative workup, as well defined in the European consensus meeting of the ARM-Net members in 2015 [17], leading to a standardized surgical procedure.

The good clinical practice of testing anal patency in newborns plays a key role in diagnosing ARMs, particularly if a normal perineum is observed. This maneuver should be routinely performed by pediatricians and neonatologists in all hospital centers in order to prevent major complications, affecting patients' morbidity and mortality.

## Data Availability

The datasets analyzed during the current study are available from the corresponding author on reasonable request.
